# Individual Differences in Inhibitory Control: A latent Variable Analysis

**DOI:** 10.5334/joc.150

**Published:** 2021-02-18

**Authors:** Anne Gärtner, Alexander Strobel

**Affiliations:** 1 Faculty of Psychology, Technische Universität Dresden, Dresden, Germany

**Keywords:** Inhibitory control, inhibition, latent variable approach, individual differences, inverse efficiency scores, confirmatory factor analysis

## Abstract

Inhibitory control represents a central component of executive functions and focuses on the ability to actively inhibit or delay a dominant response to achieve a goal. Although various tasks exist to measure inhibitory control, correlations between these tasks are rather small, partly because of the task impurity problem. To alleviate this problem, a latent variable approach has been previously applied and two closely related yet separable functions have been identified: prepotent response inhibition and resistance to distractor interference. The goal of our study was a) to replicate the proposed structure of inhibitory control and b) to extend previous literature by additionally accounting for speed-accuracy trade-offs, thereby potentially increasing explained variance in the investigated latent factors. To this end, 190 participants completed six inhibitory control tasks (antisaccade task, Stroop task, stop-signal task, flanker task, shape-matching task, word-naming task). Analyses were conducted using standard scores as well as inverse efficiency scores (combining response times and error rates). In line with previous studies, we generally found low zero-order correlations between the six tasks. By applying confirmatory factor analysis using standard reaction time difference scores, we were not able to replicate a satisfactory model with good fit to the data. By using inverse efficiency scores, a two-related-factor and a one-factor model emerged that resembled previous literature, but only four out of six tasks demonstrated significant factor loadings. Our results highlight the difficulty in finding robust inter-correlations between commonly used inhibitory control tasks, even when applying a latent variable analysis and accounting for speed-accuracy trade-offs.

## 1. Introduction

Inhibitory control represents a central component of executive functions. Although various terms and taxonomies exist, a common working definition is that inhibitory control focuses on the ability to actively inhibit or delay a dominant response to achieve a goal (Friedman & Miyake, 2004; Miyake et al., 2000; Nigg, 2000). Importantly, research has shown that inhibitory control represents a core ability that is associated with various types of executive functions, e.g., working memory updating and shifting (Miyake & Friedman, 2012). Not surprisingly, the construct is widely used in numerous research domains and has been proposed as an underlying mechanism implicated in different skills and cognitive achievements, for example attention (Friedman et al., 2007), working memory span and reading comprehension (De Beni et al., 1998; Gernsbacher, 1993), problem solving (Pasolunghi et al., 1999), general cognitive ability (Dempster & Corkill, 1999) as well as emotion regulation (Tabibnia et al., 2011). Deficient inhibition-related processes have been postulated in several forms of psychopathology and mental disorders, for example rumination (De Lissnyder et al., 2010) and depression (Joormann, 2010), externalizing behavior (Young et al., 2009), ADHD (Barkley, 1997; Nigg, 2001), substance use disorders (Nigg et al., 2006), schizophrenia (Westerhausen et al., 2011), autism (Geurts et al., 2014), and obsessive-compulsive disorder (van Velzen et al., 2014).

Despite the high relevance, some commonly used tasks to measure inhibitory control such as the Stroop task or stop-signal task often show low construct validities (Rabbitt, 1997; for a meta-analysis, see Duckworth & Kern, 2011) and poor reliabilities (Enkavi et al., 2019; recent review in Hedge et al., 2018). Furthermore, although a number of tasks have been used to tap inhibitory control, quite often only a single task is used per study (albeit for obvious reasons such as limited time and resources). Given that no tasks are pure measures of inhibitory control, it remains unclear whether the observed effects rely rather on idiosyncratic task requirements instead of inhibitory control. This well-known task impurity problem (that is related to all executive functions) indicates that, since any target inhibitory control process must be embedded in a specific context, systematic variance is attributable to non-inhibitory control abilities (Miyake et al., 2000). This and random measurement error make it difficult to purely measure inhibitory control variance. Consequently, low zero-order and often insignificant correlations between commonly used inhibitory control tasks have been reported and likely result from these problems (e.g. Enge et al., 2014; Singh et al., 2018).

As several studies pointed out previously, using multiple tasks and applying a latent variable analysis provides a more fruitful and reliable measurement of inhibitory control (e.g., Aichert et al., 2012; Miyake et al., 2000; Stahl et al., 2014). By extracting common variance that is shared by all tasks, latent variables provide purer measures, thereby reducing measurement error and the task impurity problem. In their seminal study regarding the unity and diversity of inhibition-related functions, Friedman and Miyake (2004) investigated the structure of three inhibition-related functions: *prepotent response inhibition, resistance to distractor interference* and *resistance to proactive interference*. By using structural equation modeling, the authors demonstrated that prepotent response inhibition and resistance to distractor interference are closely related to each other (*r* = .67) but separable from resistance to proactive interference. A study by Kane and colleagues (2016) confirmed the pattern that individual differences in inhibition-related functions represent distinguishable yet empirically related constructs. They found a robust association between attention restraint (e.g., antisaccade and Stroop task) and attention constraint abilities (e.g., flanker task) with a correlation (.60) similar to the study by Friedman and Miyake (.68), but also that these skills were distinguishable and not identical.

The first goal of this study was to replicate the finding by Friedman and Miyake (2004) of two latent variables (‘prepotent response inhibition’ and ‘resistance to distractor interference’) using a latent variable approach. We were especially interested whether prepotent response inhibition and resistance to distractor interference are in fact closely related, given that several studies emphasize conceptual differences between both types of inhibitory control (Dempster, 1995; Harnishfeger, 1995; Nigg, 2000) or even suggest both constructs to be empirically independent (Tiego et al., 2018). In detail, it has been postulated that resistance to distractor interference relates to an initial perceptual stage of information processing and focuses on the selection of relevant vs. irrelevant information. In contrast, prepotent response inhibition has been associated with a later stage of information processing, focusing on the inhibition of motor responses and behavioral impulses. Because of several methodological problems for resistance to proactive interference (e.g., the dependent variable in the respective tasks represents a difference score that results from only one measurement value, cf. Friedman & Miyake, 2004), we focused only on the relationship between prepotent response inhibition and resistance to distractor interference.

The second goal of the study was to account for the speed-accuracy trade-off that is inherently related to all tasks that rely on instructions that emphasize to respond as fast and as accurately as possible. However, given the inverse relationship between speed and accuracy in both animals and humans, known as the speed-accuracy trade-off (Bogacz, 2013; Wickelgren, 1977), performance measures based on either reaction times (e.g., Stroop effect) or error rates alone may be difficult to interpret (cf. Enge et al., 2014). For example, Draheim et al. (2019) extensively discussed the problems and alternatives of using standard reaction time difference scores in differential and developmental research in their recent review. Because previous work on integrated speed-accuracy measures has been based on simulation data or applied in more experimental paradigms (Heitz, 2014; Vandierendonck, 2017), this study investigates whether one such integrated measure empirically improves the measurement issue of more conventional reaction time (RT) difference scores for individual differences research. Therefore, we combined error rates and reaction times into inverse efficiency scores (IES; Bruyer & Brysbaert, 2011; Townsend & Ashby, 1983) by dividing the mean RT of correct responses by the proportion of correct responses. This was done in a previous study that applied a latent variable approach to executive function tasks (Wolff et al., 2016) and has the advantage that reaction time and accuracy are combined into a single performance measure. Specifically, we expected higher correlations and a better fit for estimated models with the IES compared to the standard outcome measures.

In sum, by using a latent variable analysis on six inhibitory control tasks, we aimed at replicating the general pattern of two closely related latent factors (cf. Friedman & Miyake, 2004): prepotent response inhibition (antisaccade task, Stroop task, stop-signal task) and resistance to distractor interference (Eriksen flanker task, shape-matching task, word-naming task; see below). In addition, we tried to extend previous literature by additionally considering speed-accuracy trade-offs using inverse efficiency scores, thereby potentially increasing explained variance in the investigated latent factors.

## 2. Methods

We report how we determined our sample size, all data exclusions (if any), all manipulations, and all measures in the study (Simmons et al., 2012). Data and analysis routines can be found at: *https://osf.io/2fwm4*.

### Participants

The sample comprised 190 healthy adults (97 female; age = 18–39 years, *M* = 23.8 years, *SD* = 4.7 years) recruited at the TU Dresden. This sample size permitted a participant-to-parameter ratio of more than five in all models (as recommended by Hatcher & O’Rourke, 2014; see also Kline, 2016). Furthermore, this sample fits about the minimum sample size for the model structure with two latent and six observed variables (cf. Cohen, 1988; Westland, 2010), at an alpha level of 0.05, a power (1–beta) of 0.80, and an anticipated effect size of 0.38 according to the initial model of inhibition related functions in Friedman and Miyake (2004; calculated with the online calculator by Soper, 2018).

In a semi-structured interview for psychiatric and neurological disorders or treatment, none of the participants reported any current or past (in the last year) medical, neurological or psychiatric illness or treatment that might influence cognition or motor performance. All participants were non-smokers, reported German as their mother tongue, had normal or corrected to normal vision and no color blindness, and reported no regular substance or alcohol use. The study design was approved by the ethics committee of the TU Dresden (EK 357092014). The study was conducted in accordance with the Declaration of Helsinki and followed the ethical guidelines of the German Psychological Association. All participants provided written informed consent and received compensation for expenses.

### Procedure

Upon arrival, participants were briefly familiarized with the laboratory setting, informed about the upcoming experiment, provided demographic information and ratings on their current mood. Afterwards, participants performed six inhibitory control tasks (see below) in randomized order. Finally, participants were debriefed, reimbursed and thanked. The session lasted approximately 90 minutes. To ensure undisturbed testing, the sessions were carried out in testing booths. Participants were allowed breaks of self-chosen duration following completion of each task inside the testing booth. To prevent that breaks were skipped completely, participants were instructed to pause by leaving the testing booth for 5 minutes after completing the first three tasks. Since circadian variation might impact on cognitive performance (Bratzke et al., 2012; Hasher et al., 1999; Schmidt et al., 2007) all sessions were conducted between 9 am and 5 pm. Because the study was part of a larger project, all participants returned for a second session investigating general emotion regulation ability. These data are not reported here. A complete list of all measures in the larger project can be found at *https://osf.io/2fwm4*.

### Measures and materials

#### Inhibitory control battery

The task battery comprised six computerized reaction time tasks, three for prepotent response inhibition (antisaccade task, Stroop task, stop-signal task) and three for resistance to distractor interference (Eriksen flanker task, shape-matching task, word-naming task). Since we followed the approach taken by other authors in previous work on individual differences in inhibitory control, the tasks were adapted from Friedman and Miyake (2004) and Enge and colleagues (2014), respectively. Whereas most of the tasks were identical to the tasks by Friedman and Miyake (2004), our implementation differed slightly with regard to the Stroop task (where we used a color-word conflict instead of number-denotation conflict), and the stop-signal task (where we used a standard response format per button press instead of an auditory version). However, as can be seen in the work by Enge et al. (2014), these tasks are equally suitable for measuring inhibitory control. All tasks were preceded by written on-screen instructions and at least 20 practice trials. A QUERTZ layout keyboard, and a microphone with audio cable, respectively, was used to enter responses. In each task, both error rate and response time were recorded.

*Antisaccade task.* During each trial of the antisaccade task (described in Friedman & Miyake, 2004), a fixation cross appeared in the middle of a white screen with a jitter of 1500–3500 ms in 250 ms intervals, followed by a visual cue on one side of the screen for 175 ms, followed by a target stimulus (an arrow inside an open box) on the opposite side of the screen for 150 ms, followed by a gray mask that remained on the screen until the participant indicated the direction of the previously shown leftward, rightward or downward pointing arrow per button press (leftward, rightward, and downward pointing arrows on the keyboard, respectively). After 22 practice trials, participants received 90 target trials.

*Stroop task.* During each trial of the classical color Stroop task (described in Enge et al., 2014), a fixation cross was presented for 500 ms on a white screen, followed by different color names (“GREEN”, “RED”, “BLUE”) or a neutral stimulus (“+ + + +”) in varying font colors (green, red, or blue) for up to 1000 ms. Participants were instructed to identify the color of the presented stimulus by button press (red: leftward pointing arrow; green: downward pointing arrow; blue: rightward pointing arrow). Three types of trials were administered: congruent trials (matched font color and word meaning), incongruent trials (mismatched font color and word meaning), and neutral trials (neutral stimulus presented in one of the font colors). The three conditions were presented intermixed in a fixed random order. After 24 practice trials, participants received 240 target trials (80 per condition).

*Stop-signal task.* During the stop-signal task (described in Enge et al., 2014), a fixation cross was presented for 500 ms on a white screen, followed by a series of black capital letters for up to 1000 ms. Participants were instructed to discriminate between vowels and consonants per button press (go trial; vowels: leftward pointing arrow, consonants: rightward pointing arrow). On the minority of trials (25%), a letter appeared in red font color or changed its color after a few milliseconds from black to red (stop signal). Here, participants were instructed to suppress their response (stop trial). The delay between the stimulus and the stop signal (stop-signal delay, SSD) varied from 0 to 500 ms in 100 ms intervals (resulting in six steps that varied randomly). We assessed the stop-signal reaction time (SSRT) as the estimated time at which the stopping process finishes. As recommended by Logan (1994) and also pursued by Friedman and Miyake (2004), we used the common estimation method based on the horse-race model with the SSRT assumed to be a constant: For each SSD, all RTs for the go trials were rank ordered. Then, the number of the SSD was subtracted from the *n*th RT, where *n* was the number of all go trial RTs multiplied with the probability of responding at that delay. After 40 practice trials, participants received 440 target trials.

*Eriksen flanker task.* During each trial of the Eriksen Flanker task (described in Friedman & Miyake, 2004), a blank white screen was presented for 1000 ms, followed by a fixation cross for 500 ms, followed by a centrally presented letter. Participants were instructed to indicate by button press whether the target letter was *H* or *K* (CTRL right) and *S* or *C* (CTRL left), respectively. The letter was presented alone (no-noise condition, “H”) or flanked by three noise letters on each side, resulting in another three conditions: 1) noise same as target (“HHHHHHH”), 2) noise compatible (“KKKHKKK”), and 3) noise incompatible (“SSSHSSS”). The stimuli remained on the screen until the participant responded. The four conditions were presented intermixed in a fixed pseudorandom order (no more than three successive trials of the same condition). After 20 practice trials, participants received 160 target trials (40 per condition).

*Shape-matching task.* During each trial of the shape-matching task (described in Friedman & Miyake, 2004; without negative priming trials), a fixation cross was presented on a black screen for 500 ms, followed by a green target shape on the left for 3000 ms (maximum), followed by a gray mask for 100 ms. Participants were instructed to indicate per button press as fast and accurately as possible whether the target shape matched (rightward pointing arrow) or mismatched (leftward pointing arrow) with a white shape on the right, ignoring the red distractor shape layering the target shape when present (distractor trial vs. no-distractor trial). A third of the trials (56) were no-distractor trials; the other 112 distractor trials. The stimuli of the task were a set of eight abstract shapes and exactly the same as used in the study by Friedman and Miyake (2004). Targets appeared equally often in each position. After 24 practice trials, participants received 168 target trials.

*Word-naming task.* During each trial of the word-naming task (described in Friedman & Miyake, 2004; without negative priming trials), a fixation cross was presented on a black screen for 500 ms, followed by a green target word on the top or bottom of the screen for 225 ms, followed by a gray mask for 100 ms, and a black screen until the participant responded. Participants were instructed to name aloud the target word and ignore the red distraction word on the opposite direction (top or bottom) when present (distractor vs. no-distractor trial). A third of the trials (56) were no-distractor trials; the other 112 distractor trials. Following the protocol by Friedman and Miyake (2004), the words were selected from eight German four-letter nouns (“TREE”, “HOUSE”, “SAND”, “RING”, “SONG”, “DOG”, “POT”, “CLOTH”, “SHIRT”), were matched in frequencies and did not rhyme. Targets appeared equally often in each position. After individual voice-key calibration and 24 practice trials, participants received 168 target trials.

### Statistical procedures

#### Data trimming and outlier analysis

In order to most closely adhere to the original analysis protocol, data trimming and outlier analysis fully followed the steps by Friedman and Miyake (2004), based on the recommendations by Wilcox and Keselman (2003) for robust data analysis. For the RT-based measures, all RTs from errors (voice key or other) and all RTs less than 200 ms were eliminated. The percentage of the trials eliminated was less than 12.5% in all of these tasks. To prevent extreme RTs unreasonably influencing the means of each participant, RTs were trimmed the following way: First, following the trimming procedure by Friedman and Miyake (2004), the following upper and lower criteria were used for each task, and any values exceeding those criteria were replaced with those values: 400 ms and 2000 ms for the Stroop task, 200 ms and 1000 ms for the word-naming task, 200 ms and 2000 ms for the shape-matching, stop-signal and antisaccade task, and 200 and 1500 ms for the flanker task. This procedure affected no more than 10% of observations for the task, except the word-naming task (33%). Second, for each participant and each task, RTs farther than 3 *SD* from the mean for each condition were replaced with the respective value 3 *SD* above/below the mean (see Wilcox & Keselman, 2003). This procedure affected no more than 2% of observations for any task. Data for the stop-signal task were not subject to this procedure because the dependent measure was not influenced by extreme RTs. Afterwards, all between-participant distributions were examined for extreme scores. For each variable used in further analyses, observations farther from 3 *SD* from the group mean were replaced with the respective value. This final trimming procedure affected no more than 2.5% of observations for any task. This data trimming procedure was set up before data analysis and aimed at closely replicating the procedure by Friedman and Miyake (2004). ***Tables 1*** and ***2*** (see “Results”) depict the descriptive statistics of the outcome measures.

**Table 1:** Descriptive statistics for response times, error rates and IES for single conditions of the six inhibitory control tasks.


VARIABLE	*M*	*SD*	RANGE

*Response Times*			

Stroop RT_incon_	655.07	119.96	445.60 to 1033.06

Stroop RT_con_	607.02	95.78	456.00 to 899.64

Antisaccade RT	457.87	127.01	239.85 to 1031.01

Stop-signal RT_go_	641.96	85.24	471.20 to 893.03

Flanker RT_incom_	593.88	105.57	338.74 to 935.57

Flanker RT_no-noise_	552.69	90.85	339.15 to 862.20

Shape-matching RT_dis_	974.07	209.74	659.41 to 1700.01

Shape-matching RT_no-dis_	808.95	121.84	598.48 to 1253.49

Word-naming RT_dis_	388.76	76.80	288.56 to 727.25

Word-naming RT_no-dis_	363.54	74.32	263.64 to 697.33

*Error Rates*			

Stroop err%_inc_	0.04	0.08	0.00 to 0.93

Stroop err%_con_	0.05	0.06	0.00 to 0.61

Antisaccade err%	0.16	0.13	0.00 to 0.66

Stop-signal err%_stop_	0.61	0.19	0.00 to 1.00

Flanker err%_incom_	0.08	0.11	0.00 to 0.50

Flanker err%_no-noise_	0.06	0.11	0.00 to 0.50

Shape-matching err%_dis_	0.06	0.10	0.00 to 0.88

Shape-matching err%_no-dis_	0.05	0.07	0.00 to 0.93

Word-naming err%_dis_	0.34	0.19	0.04 to 1.00

Word-naming err%_no-dis_	0.22	0.19	0.00 to 1.00

*Inverse Efficiency Scores*			

Stroop IES_incon_	752.06	967.26	460.48 to 13872.47

Stroop IES_con_	690.62	803.10	466.25 to 11576.93

Flanker IES_incom_	661.97	214.50	447.38 to 1871.15

Flanker IES_no-noise_	606.84	198.21	402.38 to 1724.41

Shape-matching IES_dis_	1091.97	584.52	740.63 to 7199.81

Shape-matching IES_no-dis_	886.55	578.91	619.58 to 8641.50

Word-naming IES_dis_	635.28	365.03	365.80 to 4751.04

Word-naming IES_no-dis_	485.92	209.79	323.24 to 2302.86


*Note*: RT = reaction time; err% = error rate (in percent); IES = inverse efficiency score; incon = incongruent; con = congruent; incom = incompatible; dis = distractor; no-dis = no distractor trials. Given that the stop signal reaction time (SSRT) already accounts for speed and accuracy, no IES were computed.

**Table 2:** Descriptive statistics for the RT and IES outcome measures of the six inhibitory control tasks.


MEASURE	*M*	*SD*	RANGE	SKEW	KURT	REL

RT						

*Response inhibition*						

Antisaccade (%)	14.37	11.32	0 to 59	1.74	3.86	.93

Stroop effect (ms)	47.52	51.52	–58 to 223	0.70	0.24	.61

Stop-signal RT (ms)	321.62	42.57	200 to 437	0.30	-0.08	.64

*Distractor Interference*						

Flanker effect (ms)	47.06	38.72	–69 to 177	0.61	3.07	.47

Shape effect (ms)	160.04	113.02	–11 to 638	2.40	7.02	.98

Word effect (ms)	25.23	26.90	–80 to 136	0.59	3.22	.31

IES						

*Response Inhibition*						

Antisaccade (ms)	570.3	284.7	292 to 2651	4.14	23.05	–

Stroop (ms)	49.6	52.6	–60 to 201	0.67	-0.06	–

Stop-signal (SSRT)°	321.6	42.6	200 to 437	0.30	-0.08	–

*Distractor Interference*						

Flanker (ms)	51.3	59.6	–96 to 319	0.93	2.78	–

Shape-matching (ms)	163.3	96.0	–46 to 529	1.19	1.69	–

Word-naming (ms)	131.1	91.3	–132 to 425	0.54	0.60	–


*Note*: RT = reaction time; IES = inverse efficiency scores; % = percent incorrect; Skew = skewness; Kurt = excess kurtosis; Rel = reliability calculated as internal consistency by adjusting split-half correlations with the Spearman-Brown formula; °given that the stop signal reaction time (SSRT) already accounts for speed and accuracy, no IES were computed.

To further ensure that extreme values did not influence the results, we checked for outliers and influential cases using leverage, studentized residuals, and Cook’s *D* values. These values assess the influence of a single variable on the correlations. Extreme values were defined by leverage values >.05; studentized residuals > |3.00|; and *D* much larger than for the rest of the observations. Although some observations were indicated as extreme values, the correlations did not change when these observations were removed. In addition, we report robust Spearman rank correlations because this test does not rely on any assumptions about the distribution of the data, thereby providing a more conservative measure for potential associations. In all tasks, lower scores indicate better performance.

#### Behavioral Analyses

Behavioral data were acquired using Presentation® software (Version 17.0, Neurobehavioral Systems, Inc., Berkeley, CA, *www.neurobs.com*), running at 24-inch LCD screens with a resolution of 1080 × 1024. Data were examined for normality and outliers using QQ plots and boxplots and analyzed using SPSS Statistics for Macintosh (Version 25; IBM Corp., Armok, NY, USA) and RStudio (Version 1.0.143). Multivariate normality was examined using the R package *MVN* (Korkmaz, Goksuluk, & Zararsiz, 2014).

Participants received the standard instruction to respond as fast and as accurately as possible (Enge et al., 2014; Friedman & Miyake, 2004). The dependent variables for the analyses with the standard RT differences were: 1) the proportion of errors in the antisaccade task, 2) the reaction time difference between incongruent and congruent trials in the Stroop task, 3) the SSRT in the stop-signal task, 4) the reaction time difference in the no-noise versus noise incompatible condition in the Eriksen flanker task, 5) and 6) the reaction time difference between the distractor versus no-distractor condition in the shape-matching and word-naming task, respectively.

Because of the related speed-accuracy trade-off, error rates (ERs) and RTs were combined into inverse efficiency scores (IES; Bruyer & Brysbaert, 2011; Townsend & Ashby, 1983) by dividing the mean RT of correct responses by the proportion of correct responses (RT/[1–ER]). In the antisaccade task, the mean RT of correct responses in the target trials was divided by the proportion of correct responses during these target trials. In the Stroop task, the mean RT of correct responses in the incongruent trials was divided by the proportion of correct responses during incongruent trials, and the mean RT of correct responses in congruent trials was divided by the proportion of correct responses during congruent trials, and the quotients were subtracted by each other (i.e., (RT_inc_/[1–ER_inc_]) – (RT_con_/[1–ER_con_]. In the Eriksen flanker task, the mean RT of correct responses during noise incompatible trials was divided by the proportion of correct responses during noise incompatible trials, and the mean RT of correct responses in no-noise trials was divided by the proportion of correct responses during no-noise trials, and the quotients were subtracted by each other (i.e., (RT_inc_/[1–ER_inc_]) – (RT_no-noise_/[1–ER_no-noise_]. In the shape-matching and word-naming task, the mean RT of correct responses in the distractor trials was divided by the proportion of correct responses during distractor trials, and the mean RT of correct responses in no-distractor trials was divided by the proportion of correct responses during no-distractor trials, and the quotients were subtracted by each other (i.e., (RT_dis_/[1–ER_dis_]) – (RT_no-dis_/[1–ER_no-dis_]. Because RTs are expressed in milliseconds (ms) and divided by proportions, IES are equally expressed in ms. IES were not used for the stop-signal task, because the SSRT already accounts for accuracy (cf. Logan et al., 2014 for further details). Descriptive statistics of the IES outcome measures, reaction times, error rates, and IES per condition and per task are given in ***Tables 1*** and ***2***. All analyses were conducted using both standard response time outcomes as well as IES to examine possible differences between both measures.

#### Model estimation

Models were estimated with AMOS (Arbuckle, 2014) using the maximum likelihood (ML) estimation based on the covariance matrix (cf. Friedman & Miyake, 2004). As a prerequisite of ML estimation, we checked multivariate normality with Mardia’s coefficient and Mahalanobis *d*^2^. Mardia’s coefficient of multivariate skewness and kurtosis was significant and several multivariate outliers were indicated by significant Mahalanobis *d*^2^ values. The results were the same when these outliers were removed. For this reason, all subjects were included in further analyses. Nevertheless, to critically evaluate the stability of parameter estimates, we bootstrapped the data 5000 times non-parametrically with replacement. This has been shown to generate less biased estimates compared to standard ML estimation for sample sizes around *N* = 200 (Nevitt & Hancock, 2001) with only moderate skewness (≤ 2) and kurtosis (≤ 7) (Gao et al., 2008). Bias-corrected standard errors and *p*-values were obtained by bootstrapping with *N* = 5000 samples (see Supplementary Table A1).

Model fit was evaluated using multiple indices according to the recommendation of Hu and Bentler (1999): chi-square statistic, the standardized root mean square residual (SRMR), the root mean square error of approximation (RMSEA), Bentler’s comparative fit index (CFI), and the normed fit index (NFI). In addition, Akaike’s information criterion (AIC) was examined (Burnham & Anderson, 2003). The chi-square statistic measures the “badness of fit” of the model compared with a saturated model, that is, the degree to which the covariances predicted by the model differ from the observed covariances (small values indicate no statistically meaningful differences and are therefore preferable). Compared to chi-square, the AIC takes the model complexity into account and was used to compare different models in order to determine the most adequate one (models yielding the lowest AIC are preferred). SRMR is an index of the average of standardized residuals between the observed and the predicted covariance matrixes; lower values indicate closer fit, values less than .08 indicate fair fit and less than .05 indicate good fit. RMSEA is an index of the difference between the observed covariance matrix per degree of freedom and the hypothesized covariance matrix which denotes the model (Chen, 2007). It also takes model complexity into account; lower values indicate closer fit, values less than .08 indicate an acceptable fit, less than .05 good fit, and less than .01 excellent fit. The CFI quantifies the extent to which the model is better than a baseline model (e.g., with covariances set to 0), and values above .95 indicate good fit, although .90 is also commonly used. The NFI measures the discrepancy between the chi-squared value of the hypothesized model and the chi-squared value of the null model; values above .95 indicate good fit. All analyses used an alpha level of .05.

## 3. Results

***Table 1*** depicts the descriptive statistics for response times, error rates and IES for single conditions of the six inhibitory control tasks.

As shown in ***Table 2***, the reliability estimates for the outcome measures of the six inhibitory control tasks were only moderate (high for antisaccade and shape-matching task, moderate for stop-signal and Stroop task, and low for flanker and word-naming task).

Inter-correlations of reaction times and error rates for the six inhibitory control tasks are depicted in ***Table 3***. There were mostly significant positive correlations between mean reaction times and error rates among the tasks. For example, Stroop mean RT correlated with stop-signal reaction time (SSRT) and antisaccade mean RT, and Stroop error rate correlated with stop-signal error rate and antisaccade error rate (see ***Table 3***).

**Table 3:** Inter-correlations of reaction times and error rates for the six inhibitory control tasks.


Variable	1	2	3	4	5	6	7	8	9	10	11

1 Stroop RT	—										

2 Antisaccade RT	.36***	—									

3 Stop-signal RT	.26***	.21**	—								

4 Flanker RT	.61***	.48***	.29***	—							

5 Shape-matching RT	.61***	.48***	.18*	.55***	—						

6 Word-naming RT	.18*	.10	.13°	.16*	.17*	—					

7 Stroop err%	.03	–.123°	–.18*	–.13°	–.13°	–.02	—				

8 Antisaccade err%	.03	.38***	–.03	.14*	.13°	–.02	.18*	—			

9 Stop-signal err%	–.14°	–.08	–.90***	–.17*	–.04	–.11	.15*	.09	—		

10 Flanker err%	–.19*	–.07	–.10	–.15*	–.15*	–.06	.21**	.25**	.13°	—	

11 Shape-matching err%	–.17*	–.17*	–.08	–.16*	–.20**	.01	.29***	.19**	.07	.19**	—

12 Word-naming err%	.12°	.11	–.13°	.12°	.16*	–.39***	.02	.27***	.16*	.04	.05


*Note*: RT = mean reaction time; err% = error rate (in percent) in stop/distractor/incongruent trials; ° *p* < .10; * *p* < .05; ** *p* < .01; *** *p* < .001.

Bivariate zero-order correlations between the tasks are shown in ***Table 4***. The magnitudes of these correlations were generally low (.29 or smaller). Using standard reaction time scores, there were significant positive correlations between performance in the antisaccade task and the stop-signal task and between performance in the Stroop task and the shape-matching task. Furthermore, there were correlations with *p* < .10 between performance in the Stroop task and the stop-signal task and between performance in the flanker task and the shape-matching task. Using IES, there were still correlations between performance in the antisaccade task and the stop-signal task and between performance in the Stroop task and the shape-matching task, and a correlation with *p* < .10 between performance in the Stroop task and the stop-signal task. Compared to the standard reaction time scores, there were now significant correlations between performance in the antisaccade task and the shape-matching task as well as performance in the antisaccade task and the word-naming task. Furthermore, there was a correlation with *p* < .10 between performance in the stop-signal task and the word-naming task.

**Table 4:** Spearman correlations of the six inhibitory control tasks for RT scores and IES.


	1		2		3		4		5	
				
	RT^a^	IES	RT	IES	RT	IES	RT	IES	RT	IES

1 Antisaccade										

2 Stroop	.07	.11								

3 Stop-signal	.16*	.19**	.13	.13						

4 Flanker	–.03	–.02	.03	.10	–.05	–.11				

5 Shape-matching	.12	.18*	.29***	.21**	–.06	–.08	.14	.06		

6 Word-naming	.00	.18*	.05	.03	–.07	.13	.05	.11	.01	.11


*Note*: RT indicates correlations between reaction time measures with the exception of ^a^ where error rates in percent in the antisaccade task were correlated with reaction time measures in the other tasks; IES indicates correlations between inverse efficiency scores. Correlations between RT and IES are not shown. * *p* < .05; ** *p* < .01; *** *p* < .001.

### The two-factor model of the inhibition-related functions

**Figure 1:**
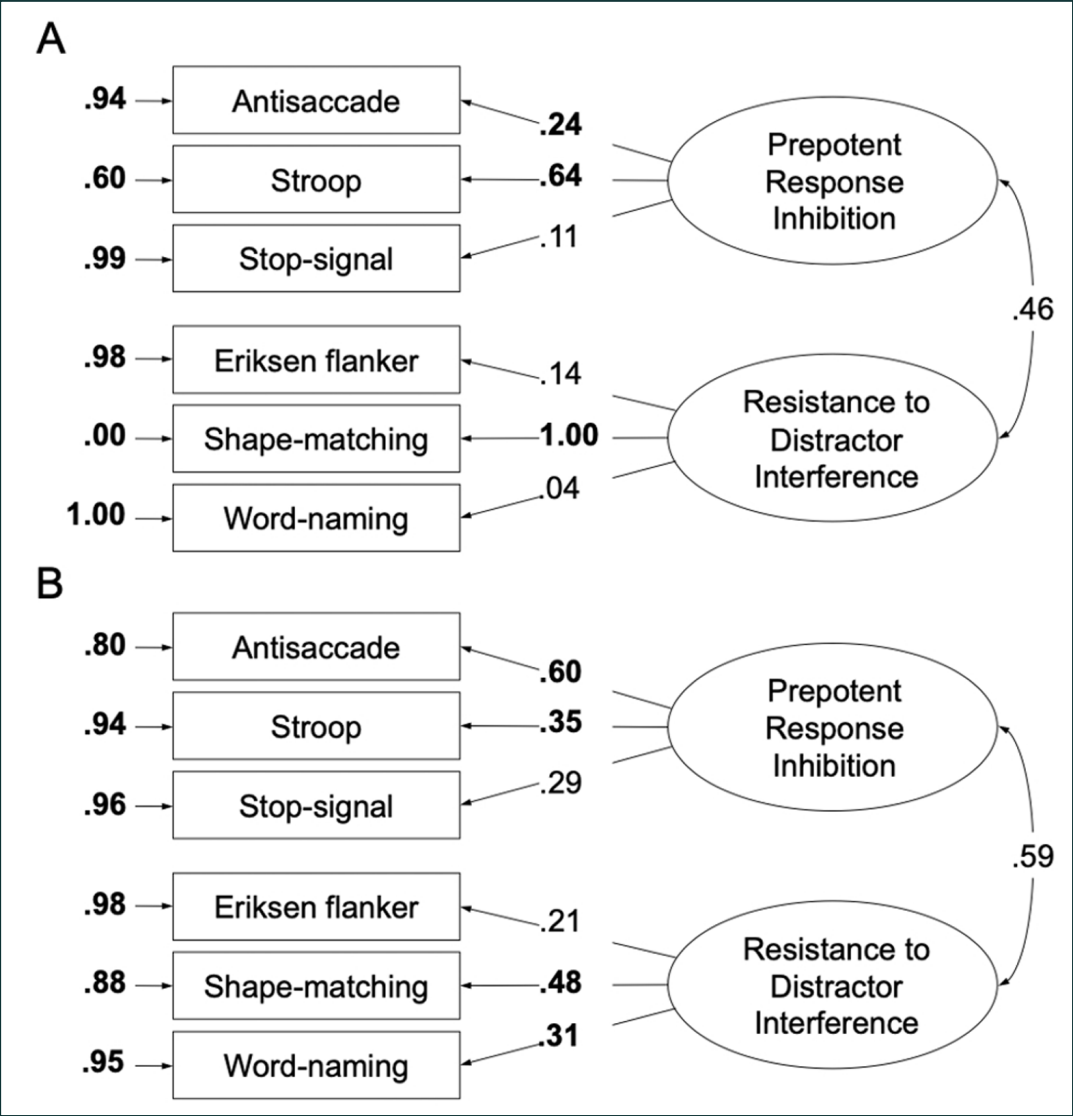
The two-factor model of inhibition-related functions using RT scores **(A)** and inverse efficiency scores **(B)**, completely standardized solution. Numbers on the leftwards single-headed arrows are standardized factor loadings. Numbers on the rightwards smaller arrows depict error variances for each task, attributable to idiosyncratic task requirements and measurement error. The number on the curved double-headed arrow is the correlation between the latent variables. Bold-face type indicates significance at the .05 level.

We constructed the measurement model of the two inhibition-related functions for both RT scores (***Figure 1A***) and IES (***Figure 1B***). ***Table 5*** also presents the fit of the null model (all covariances among the tasks are hypothesized to equal zero, but variances of the tasks are allowed to vary freely). Given the low zero-order correlations between the tasks, one might speculate that there is not much to be modelled and that the fit of any model would be adequate. However, the fit of the null model was poor, 𝘟^2^(15, *N* = 190) = 40.12, *p* < .001, RMSEA = .094, SRMR = .090, CFI < .01, AIC = 64.12, NFI < .01. Therefore, the covariances are substantial enough to support model-fitting procedures. As shown in ***Table 5***, the fit of the depicted model with two related factors (prepotent response inhibition and resistance to distractor interference) was poor, 𝘟^2^(8, *N* = 190) = 12.65, *p* > .05, RMSEA = .055, SRMR = .053, CFI = .82, AIC = 50.65, NFI = .685. Furthermore, only three tasks demonstrated significant factor loadings (antisaccade task, Stroop task, shape-matching task) and the two factors were not significantly related to each other. ***Table 5*** also presents the fit statistics for alternative theoretical models that we considered (two factors unrelated, one factor). The fit statistics of these models were comparable (one factor) or even worse (two factors unrelated). Supplementary Table A1 contains the bootstrapped *p*-values and standard errors of the depicted model in ***Figure 1***. Note that the fit of all models was evaluated according to the fit criteria reported in ***Table 5***.

**Table 5:** Fit statistics for the confirmatory factor analysis models.


Model	*df*	*x*^2^	RMSEA	SRMR	CFI	AIC	NFI

*Reaction time scores*							

Null model	15	40.12***	.094	.090	.000	64.115	.000

Two factors unrelated	9	26.48**	.101	.079	.304	62.476	.340

Two factors related	8	12.65	.055	.053	.815	50.645	.685

One factor	9	15.34	.061	.057	.748	51.340	.618

*Inverse efficiency scores*							

Two factors unrelated	9	19.71*	.079	.074	.619	55.707	.543

Two factors related	8	11.62	.049	.049	.871	49.623	.730

One factor	9	14.03	.054	.041	.821	50.030	.674


*Note*: Reasonable fit: Chi-squares not significant at the .05 level; lower values of root mean square error of approximation (RMSEA) with <.08 mediocre fit, <.05 good fit and <.01 excellent fit; lower values of standardized root-mean-square residual (SRMR) with <.08 fair fit, <.05 good fit; values above .95 for Bentler’s comparative fit index (CFI) for excellent fit; lower values of Akaike’s information criterion (AIC); values above .95 for the normed fit index (NFI) for good fit * p < .05.

As mentioned earlier, we constructed the same measurement models with IES and expected better model fit by taking reaction time and accuracy for each task into account. The fit for the model with two related factors was mediocre, 𝘟^2^(8, *N* = 190) = 11.62, *p* > .05, RMSEA = .049, SRMR = .049, CFI = .87, AIC = 49.62, NFI = .730. The fit was preferable over the model with unrelated factors but comparable to the model with one factor. However, there were still two tasks that demonstrated no significant factor loadings (stop-signal task, flanker task).

## 4. Discussion

In this study, we examined the relationship between six commonly used inhibitory control tasks and aimed at replicating the general pattern of two closely related latent variables (prepotent response inhibition, resistance to distractor interference). In addition, well-known speed-accuracy trade-offs were taken into account by considering inverse efficiency scores (IES). In line with previous studies (Aichert et al., 2012; Cheung et al., 2004; Enge et al., 2014; Enticott et al., 2006), we found generally low and non-significant zero-order correlations between the six tasks. By using standard reaction time difference scores, we were not able to replicate a satisfactory latent variable model with good fit to the data. In contrast, by using IES, both a two-related and a one-factor model with the latent variable response–distractor inhibition indicated mediocre fit to the data and resembles previous literature (Friedman & Miyake, 2004), although only four out of six tasks demonstrated significant factor loadings. The results highlight the difficulty in finding robust inter-correlations between inhibitory control tasks, even when accounting for speed-accuracy trade-offs, thereby possibly reflecting the consequence of the task impurity problem.

The magnitudes of the correlations between the six inhibitory control tasks were generally low (.29 or smaller), but are consistent with the results of previous studies and seem not to be restricted to college samples, but also present in samples with a wider age range and across different levels of intellectual abilities (Cheung et al., 2004; Enge et al., 2014; Enticott et al., 2006; Friedman & Miyake, 2004; Miyake et al., 2000; Shilling, Chetwynd, & Rabbitt, 2002; Singh et al., 2018; Wolff et al., 2016). This is why we applied a latent variable analysis: By extracting common variance that is shared by all tasks, latent variables provide purer measures, thereby reducing measurement error and the task impurity problem. Indeed, the fit for the null model (assuming that the covariances among all tasks are zero) was poor, indicating that although we found only low and mostly non-significant zero-order correlations, the covariances did support model-fitting procedures. However, applying the commonly used reaction time difference scores for the measurement model, we were not able to find a satisfactory fit for the two-factor model (prepotent response inhibition and resistance to distractor interference) or the alternative one-factor model (response–distractor inhibition) based on the findings of Friedman and Miyake (2004). Only three tasks demonstrated significant factor loadings (antisaccade task, Stroop task, shape-matching task).

Given that participants are generally instructed to respond as fast and accurately as possible when conducting these or similar executive function tasks, speed-accuracy trade-offs are likely to be expected. Indeed, negative correlations between mean reaction times and error rates were observed in our study, indicating enhanced speed at the expense of accuracy (see ***Table 3*** for further details). Therefore, in a second step we computed a composite score combining reaction times and error rates in a single score (IES). Using IES, inter-correlations between tasks remained mostly the same (positive correlation between performance in the shape-matching and the Stroop task, as well as between the stop-signal and the antisaccade task) and two additional correlations were observed (positive correlations between the antisaccade task and the shape-matching and word-naming task, respectively). When constructing the measurement model, the fit for the model with two related factors was only moderate and two tasks still demonstrated non-significant factor loadings (stop-signal task, flanker task).

The failure to extract a satisfactory inhibitory control factor using latent variable analysis is consistent with a line of other studies (e.g., Friedman & Miyake, 2017; Huizinga et al., 2006; Logan et al., 2014; Singh et al., 2018; van der Sluis et al., 2007). Given the generally low zero-order correlations, low factor loadings and high amount of unexplained variance (77–95%), one might conclude that the task measures for inhibitory control used in our and in other studies make it difficult to reliably measure a latent factor. This likely reflects the task impurity problem, that is, the fact that systematic variance is attributable to non-inhibitory abilities (e.g., specific task demands, differing task properties, measurement error). However, similar results regarding low zero-order correlations and factor loadings have also been found for tasks measuring working memory updating and shifting ability (Friedman & Miyake, 2017; Huizinga et al., 2006; van der Sluis et al., 2007), but these studies were able to successfully apply a latent variable approach and found higher factor loadings for the respective tasks. Therefore, another interpretation might be that, in contrast to updating and shifting, inhibitory control represents no common process. This assumption is supported by recent studies from Rey-Mermet and colleagues (2018) and Morra and colleagues (2018), emphasizing that the inhibition construct may need to be separated into different subtypes (see also Noreen & MacLeod, 2015). Instead, studies investigating inhibitory control as a latent variable often found that most of the variance can be accounted for by another factor, that is, basic naming speed (a non-executive processing demand in verbal tasks) and goal maintenance, respectively (Singh et al., 2018; van der Sluis et al., 2007; Friedman & Miyake, 2017). Although goal maintenance is a crucial prerequisite in all executive function tasks, it may be particularly important for inhibition tasks in which the main requirement is avoiding strong prepotent responses or conflicting information. This mechanism could explain why inhibitory control tasks often load on a common executive function factor, but not on an additional inhibition specific factor (Friedman & Miyake, 2017). However, the issue is likely more complicated, given that we found no latent factor (representing goal maintenance) for all tasks. Clearly, more research is needed to disentangle the effects of specific task demands (e.g., by using multiple versions of the same task), inhibitory control ability, and other involved processes like attention and basic naming speed.

A comprehensive study by Stahl et al. (2014) investigated behavioral components of impulsivity, among them resistance to distractor interference and prepotent response inhibition (which they called “stimulus interference” and “behavioral inhibition”, respectively). In contrast to our findings, they were able to find two latent factors for both constructs using a structural equation modeling approach, but they were not significantly correlated (as opposed to Friedman & Miyake, 2004). The authors argued that this could be attributable to the applied tasks: In their view, the Stroop task and the flanker task involve both distractor- and response-related interference, which might possibly reduce the amount of ability-specific variance in the respective latent factors. This is also in line with the study by Tiego et al. (2018), who classify the Stroop task among the flanker and shape-matching task as measures for distractor interference. Following this line of reasoning, the proposed unitary nature of the response-distractor inhibition factor might be artefactual and possibly reflects a failure to use appropriate tasks, or task modifications, to circumvent the task impurity problem. Indeed, this interpretation seems to be partly supported by our data, with the strongest zero-order correlation observed between performance in the Stroop task and the shape-matching task (*r* = .29, *p* < .001), a correlation that would be expected if both were measuring resistance to distractor interference. Interestingly, similar correlations were found in the studies of Stahl et al. (2014; *r* = .21, *p* < .05) and Tiego et al. (2018; *r* = .299, *p* < .01). Furthermore, the study by Tiego et al. (2018) demonstrated that resistance to distractor interference and prepotent response inhibition were empirically unrelated when individual differences in working memory capacity were taken into account. Although the study was carried out in a developmental sample, it shows that the empirical overlap of both inhibitory control concepts might at least partly be explained by their common reliance on a limited-capacity attentional resource.

It should be noted that although we found generally low zero-order correlations between the tasks, there were mostly positive significant correlations between mean reaction times and error rates among the tasks. For example, Stroop mean RT correlated with stop-signal reaction time and antisaccade mean RT, and Stroop error rate correlated with stop-signal error rate and antisaccade error rate (see ***Table 3*** for further information). The fact that error rate and mean RT were correlated in nearly all tasks provides support that the errors reflect an inability to inhibit prepotent responses and distractors, respectively, and that the mean RTs reflect general impulsivity. In contrast, the difference scores (e.g., Stroop effect, flanker effect) were not correlated. This is in line with research showing that difference scores are generally lower in reliability than their components. For example, Hedge and colleagues (2018) demonstrated that the total amount of variance is reduced in difference scores often by a factor of 3 or 4 relative to their components. Therefore, the authors concluded that “robust experimental effects do not necessarily translate to optimal methods of studying individual differences” (p.17), partly because experimental designs have been developed for providing robust effects, which means low between-participant variance (Hedge et al., 2018; see also Draheim, Mashburn, Martin, & Engle, 2019; and Liesefeld & Janczyk, 2019). Furthermore, the reliance on IES has also been debated, as Bruyer and Brysbaert showed that IES increase the variability of the measure when the respective error rate of the task exceeds 10 percent. This has a critical impact on the power of the experiment (Bruyer & Brysbaert, 2011). It remains to be seen whether current alternative statistical and methodological approaches, for example, reliance on accuracy-based measures (Draheim, Tsukahara, Martin, Mashburn, & Engle, 2019) or accounting for trial-by-trial variability (Rouder & Haaf, 2019), will prove promising. For example, Draheim et al. (2019) found that accuracy-based measures improve reliability and validity of attention measures. Using a hierarchical regression model, Rouder and Haaf (2019) showed improved reliability (but not validity). Similarly, Rey-Mermet et al. (2019) attempted to reduce variance associated with general processing speed when using difference scores.

### Limitations and future directions

Although all inhibitory control tasks were adopted from Friedman and Miyake (2004), there were some variations compared to their study (e.g., Stroop task with color-word conflict instead of number-denotation conflict; stop-signal task with standard response format per button press instead of an auditory version and without tracking method). At least regarding the Stroop task, this might explain why our mean Stroop effect was approximately 100 ms smaller (147 vs. 48 ms; stop-signal reaction time was comparable with 370 vs. 332 ms). However, as we have shown previously, the tasks are equally suitable for measuring inhibitory control (Enge et al., 2014) as they still provide meaningful interference effects. Therefore, these differences in implementation did not have a substantial effect on the results. However, a limitation might arise from the comparably low reliabilities of the word-naming and flanker tasks (.31 and .47, respectively). Although we used the same number of trials as Friedman and Miyake (2004) and wanted to stay as close as possible to their protocol, 40 trials per condition are few and might have contributed to the non-significant factor loadings. The word-naming task had more trials (168), but many had to be excluded during the trimming procedure (mostly due to technical artifacts with the microphone). Therefore, further studies should include a sufficiently large (as large as possible) number of trials to enhance reliability of the tasks.

A further limitation related to the antisaccade task is that because no eye-tracker was used in the study, we cannot rule out that direction errors were missed or wrongly detected. Furthermore, the visual angle was only about 2°. A study by Kane et al. (2001) has shown that a larger visual angle (around 11°) produces more reliable results. However, at least the general error rate is comparable to other studies (e.g., Friedman & Miyake, 2004).

With a sample size of 190, the present study also meets stricter criteria for a case-to-parameter ratio of 10-20:1 instead of 5:1 (Kline, 2016). However, this sample size may still be insufficient when applying χ2 difference tests to decide between competing models with few degrees of freedom (Kenny et al., 2015). Although we wanted to stay as close as possible to Friedman and Miyake’s latent variable analyses, further studies might apply Monte Carlo simulations (e.g., Muthén & Muthén, 2002) for determining adequate sample sizes for model comparisons. A larger sample size (>250) would also benefit the examination of robust intercorrelations (e.g., see Schönbrodt & Perugini, 2013).

Another general limitation of studies like ours regards sample composition. By investigating young healthy adults in an academic setting (students), it is possible that their general cognitive control ability is already in the upper range compared to the general population or clinical samples (e.g., patients with ADHD), resulting in relatively homogenous inhibitory control performance. This could make it even more difficult to find reliable interindividual differences and potentially underestimate the effect size. In contrast, it is reasonable to speculate that individual differences in inhibition could be found in clinical samples, or can be used to distinguish between clinical and non-clinical samples. Further studies should compare different samples, for example adults of the general population and clinical patients, in order to enhance heterogeneity in the cognitive control measures (but see Rey-Mermet et al., 2018, who studied inhibitory control in young and old adults but still found only weak evidence for inhibition as a psychometric construct).

## Conclusion

In sum, our inhibition measures correlated only weakly. By accounting for speed-accuracy trade-offs using inverse efficiency scores, we were able to extract a two-related-factor and a one-factor model, respectively, but only four out of six tasks demonstrated significant factor loadings in these models. Together, these results add to the growing body of research that calls into question whether individual differences in inhibitory control can be measured reliably and validly with the existing tasks. Future studies need to generate and test specific predictions on task demands, and think of alternative measures than difference scores when investigating individual differences, or develop new tasks that are able to tap more inhibition-related variance. Otherwise, the concept of inhibitory control as a common process may no longer withstand (cf. Noreen & MacLeod, 2015).

## Data Accessibility Statement

The dataset analyzed for this study and the analysis code can be found at the Open Science Framework [*https://osf.io/2fwm4*].

## Appendix

**Supplementary Table A1:** Factor loadings and bootstrapped estimates for the respective models.

MODEL	STANDARDIZED FACTOR LOADING°	MEAN STANDARDIZED FACTOR LOADING	BIAS	*SE*	*p*

*Two factors, related (reaction time scores)*					

Antisaccade ← response inhibition	.239	.238	.000	.18	.046

Stroop ← response inhibition	.635	.662	.027	.27	.001

Stop-signal ← response inhibition	.112	.138	.025	.14	.583

Eriksen flanker ← distractor interference	.142	.149	.149	.10	.111

Shape-matching ← distractor interference	1.00	.947	.947	.16	<.001

Word-naming ← distractor interference	.038	.036	.036	.13	.619

*One factor, 4 tasks (inverse efficiency scores)*					

Antisaccade ← response distractor interference	.417	.417	.000	.16	.001

Stroop ← response distractor interference	.394	.382	–.013	.15	.007

Shape-matching ← response distractor interference	.476	.508	.033	.18	.002

Word-naming ← response distractor interference	.212	.214	.002	.13	.044

*Note*: ° without bootstrap; Bias: difference between original estimate and bootstrap mean estimate; bootstrap with *N* = 5000.

## References

[1] Aichert, D. S., Wöstmann, N. M., Costa, A., Macare, C., Wenig, J. R., Möller, H.-J., Rubia, K., & Ettinger, U. (2012). Associations between trait impulsivity and prepotent response inhibition. Journal of Clinical and Experimental Neuropsychology, 34(10), 1016–1032. DOI: 10.1080/13803395.2012.706261 22888795

[2] Arbuckle, J. L. (2014). Amos (Version 23.0) [Computer Program]. IBM SPSS.

[3] Barkley, R. A. (1997). Behavioral inhibition, sustained attention, and executive functions: Constructing a unifying theory of ADHD. Psychological Bulletin, 121(1), 65–94. DOI: 10.1037/0033-2909.121.1.65 9000892

[4] Bogacz, R. (2013). Speed-accuracy trade-off In D. Jaeger & R. Jung (Eds.), Encyclopedia of computational neuroscience (pp. 1–4). New York: Springer New York DOI: 10.1007/978-1-4614-7320-6_319-1

[5] Bratzke, D., Steinborn, M. B., Rolke, B., & Ulrich, R. (2012). Effects of sleep loss and circadian rhythm on executive inhibitory control in the Stroop and Simon tasks. Chronobiology International, 29(1), 55–61. DOI: 10.3109/07420528.2011.635235 22217101

[6] Bruyer, R., & Brysbaert, M. (2011). Combining speed and accuracy in cognitive psychology: Is the Inverse Efficiency Score (IES) a better dependent variable than the mean Reaction Time (RT) and the Percentage of Errors (PE)? Psychologica Belgica, 51(1), 5–13. DOI: 10.5334/pb-51-1-5

[7] Burnham, K. P., & Anderson, D. R. (2003). Model selection and multimodel inference: A practical information-theoretic approach. Springer https://books.google.de/books?id=BQYR6js0CC8C

[8] Chen, F. F. (2007). Sensitivity of goodness of fit indexes to lack of measurement invariance. Structural Equation Modeling: A Multidisciplinary Journal, 14(3), 464–504. DOI: 10.1080/10705510701301834

[9] Cheung, A. M., Mitsis, E. M., & Halperin, J. M. (2004). The relationship of behavioral inhibition to executive functions in young adults. Journal of Clinical and Experimental Neuropsychology, 26(3), 393–404. DOI: 10.1080/13803390490510103 15512928

[10] Cohen, J. (1988). Statistical power analysis for the behavioral sciences (2nd ed.). Lawrence Earlbaum Associates.

[11] De Beni, R., Palladino, P., Pazzaglia, F., & Cornoldi, C. (1998). Increases in intrusion errors and working memory deficit of poor comprehenders. The Quarterly Journal of Experimental Psychology Section A, 51(2), 305–320. DOI: 10.1080/713755761 9621841

[12] De Lissnyder, E., Koster, E. H. W., Derakshan, N., & De Raedt, R. (2010). The association between depressive symptoms and executive control impairments in response to emotional and non-emotional information. Cognition & Emotion, 24(2), 264–280. DOI: 10.1080/02699930903378354

[13] Dempster, F. N. (1995). Interference and inhibition in cognition: An historical perspective In Interference and inhibition in cognition (pp. 3–26). Elsevier DOI: 10.1016/B978-012208930-5/50002-7

[14] Dempster, F. N., & Corkill, A. J. (1999). Individual differences in susceptibility to interference and general cognitive ability. Acta Psychologica, 101(2–3), 395–416. DOI: 10.1016/S0001-6918(99)00013-X

[15] Draheim, C., Mashburn, C. A., Martin, J. D., & Engle, R. W. (2019). Reaction time in differential and developmental research: A review and commentary on the problems and alternatives. Psychological Bulletin, 145(5), 508–535. DOI: 10.1037/bul0000192 30896187

[16] Draheim, C., Tsukahara, J. S., Martin, J., Mashburn, C., & Engle, R. (2019). A toolbox approach to improving the measurement of attention control. PsyArXiv. DOI: 10.31234/osf.io/q985d 32700925

[17] Duckworth, A. L., & Kern, M. L. (2011). A meta-analysis of the convergent validity of self-control measures. Journal of Research in Personality, 45(3), 259–268. DOI: 10.1016/j.jrp.2011.02.004 21643479 PMC3105910

[18] Enge, S., Behnke, A., Fleischhauer, M., Küttler, L., Kliegel, M., & Strobel, A. (2014). No evidence for true training and transfer effects after inhibitory control training in young healthy adults. Journal of Experimental Psychology: Learning, Memory, and Cognition, 40(4), 987–1001. DOI: 10.1037/a0036165 24707778

[19] Enkavi, A. Z., Eisenberg, I. W., Bissett, P. G., Mazza, G. L., MacKinnon, D. P., Marsch, L. A., & Poldrack, R. A. (2019). Large-scale analysis of test–retest reliabilities of self-regulation measures. Proceedings of the National Academy of Sciences, 116(12), 5472–5477. DOI: 10.1073/pnas.1818430116 PMC6431228 30842284

[20] Enticott, P. G., Ogloff, J. R. P., & Bradshaw, J. L. (2006). Associations between laboratory measures of executive inhibitory control and self-reported impulsivity. Personality and Individual Differences, 41(2), 285–294. DOI: 10.1016/j.paid.2006.01.011

[21] Friedman, N. P., Haberstick, B. C., Willcutt, E. G., Miyake, A., Young, S. E., Corley, R. P., & Hewitt, J. K. (2007). Greater attention problems during childhood predict poorer executive functioning in late adolescence. Psychological Science, 18(10), 893–900. DOI: 10.1111/j.1467-9280.2007.01997.x 17894607

[22] Friedman, N. P., & Miyake, A. (2004). The relations among inhibition and interference control functions: A latent-variable analysis. Journal of Experimental Psychology: General, 133(1), 101–135. DOI: 10.1037/0096-3445.133.1.101 14979754

[23] Friedman, N. P., & Miyake, A. (2017). Unity and diversity of executive functions: Individual differences as a window on cognitive structure. Cortex, 86, 186–204. DOI: 10.1016/j.cortex.2016.04.023 27251123 PMC5104682

[24] Gao, S., Mokhtarian, P. L., & Johnston, R. A. (2008). Non-normality of data in structural equation models. UC Berkeley: University of California Transportation Center https://escholarship.org/uc/item/11q0s48s

[25] Gernsbacher, M. A. (1993). Less skilled readers have less efficient suppression mechanisms. Psychological Science, 4(5), 294–298. DOI: 10.1111/j.1467-9280.1993.tb00567.x 25309046 PMC4191741

[26] Geurts, H. M., van den Bergh, S. F. W. M., & Ruzzano, L. (2014). Prepotent response inhibition and interference control in autism spectrum disorders: Two meta-analyses. Autism Research, 7(4), 407–420. DOI: 10.1002/aur.1369 24596300

[27] Harnishfeger, K. K. (1995). The development of cognitive inhibition In Interference and inhibition in cognition (pp. 175–204). Elsevier DOI: 10.1016/B978-012208930-5/50007-6

[28] Hasher, L., Zacks, R. T., & May, C. P. (1999). Inhibitory control, circadian arousal, and age In D. Gopher & A. Koria (Eds.), Attention & performance, XVII, cognitive regulation of performance: Interaction of theory and application (pp. 653–675). MA: MIT Press.

[29] Hatcher, L., & O’Rourke, N. (2014). A step-by-step approach to using SAS for factor analysis and structural equation modeling. SAS Institute.

[30] Hedge, C., Powell, G., & Sumner, P. (2018). The reliability paradox: Why robust cognitive tasks do not produce reliable individual differences. Behavior Research Methods, 50(3), 1166–1186. DOI: 10.3758/s13428-017-0935-1 28726177 PMC5990556

[31] Heitz, R. P. (2014). The speed-accuracy tradeoff: History, physiology, methodology, and behavior. Frontiers in Neuroscience, 8 DOI: 10.3389/fnins.2014.00150 PMC4052662 24966810

[32] Hu, L., & Bentler, P. M. (1999). Cutoff criteria for fit indexes in covariance structure analysis: Conventional criteria versus new alternatives. Structural Equation Modeling: A Multidisciplinary Journal, 6(1), 1–55. DOI: 10.1080/10705519909540118

[33] Huizinga, M., Dolan, C. V., & van der Molen, M. W. (2006). Age-related change in executive function: Developmental trends and a latent variable analysis. Neuropsychologia, 44(11), 2017–2036. DOI: 10.1016/j.neuropsychologia.2006.01.010 16527316

[34] Joormann, J. (2010). Cognitive inhibition and emotion regulation in depression. Current Directions in Psychological Science, 19(3), 161–166. DOI: 10.1177/0963721410370293

[35] Kane, M. J., Bleckley, M. K., Conway, A. R. A., & Engle, R. W. (2001). A controlled-attention view of working-memory capacity. Journal of Experimental Psychology: General, 130(2), 169–183. DOI: 10.1037/0096-3445.130.2.169 11409097

[36] Kane, M. J., Meier, M. E., Smeekens, B. A., Gross, G. M., Chun, C. A., Silvia, P. J., & Kwapil, T. R. (2016). Individual differences in the executive control of attention, memory, and thought, and their associations with schizotypy. Journal of Experimental Psychology: General, 145(8), 1017–1048. DOI: 10.1037/xge0000184 27454042 PMC4965188

[37] Kenny, D. A., Kaniskan, B., & McCoach, D. B. (2015). The performance of RMSEA in models with small degrees of freedom. Sociological Methods & Research, 44(3), 486–507. DOI: 10.1177/0049124114543236

[38] Kline, R. B. (2016). Principles and practice of structural equation modeling (Fourth edition). The Guilford Press.

[39] Korkmaz, S., Goksuluk, D., & Zararsiz, G. (2014). MVN: An R package for assessing multivariate normality. The R Journal, 6(2), 151–162. DOI: 10.32614/RJ-2014-031

[40] Liesefeld, H. R., & Janczyk, M. (2019). Combining speed and accuracy to control for speed-accuracy trade-offs(?). Behavior Research Methods, 51(1), 40–60. DOI: 10.3758/s13428-018-1076-x 30022459

[41] Logan, G. D. (1994). On the ability to inhibit thought and action: A user’s guide to the stop signal paradigm In D. Dagenbach & T. H. Carr (Eds.), Inhibitory processes in attention, memory, and language (pp. 189–239). Academic Press.

[42] Logan, G. D., Van Zandt, T., Verbruggen, F., & Wagenmakers, E.-J. (2014). On the ability to inhibit thought and action: General and special theories of an act of control. Psychological Review, 121(1), 66–95. DOI: 10.1037/a0035230 24490789

[43] Miyake, A., & Friedman, N. P. (2012). The nature and organization of individual differences in executive functions: Four general conclusions. Current Directions in Psychological Science, 21(1), 8–14. DOI: 10.1177/0963721411429458 22773897 PMC3388901

[44] Miyake, A., Friedman, N. P., Emerson, M. J., Witzki, A. H., Howerter, A., & Wager, T. D. (2000). The unity and diversity of executive functions and their contributions to complex “frontal lobe” tasks: A latent variable analysis. Cognitive Psychology, 41(1), 49–100. DOI: 10.1006/cogp.1999.0734 10945922

[45] Morra, S., Panesi, S., Traverso, L., & Usai, M. C. (2018). Which tasks measure what? Reflections on executive function development and a commentary on Podjarny, Kamawar, and Andrews (2017). Journal of Experimental Child Psychology, 167, 246–258. DOI: 10.1016/j.jecp.2017.11.004 29197781

[46] Muthén, L. K., & Muthén, B. O. (2002). How to use a monte carlo study to decide on sample size and determine power. Structural Equation Modeling: A Multidisciplinary Journal, 9(4), 599–620. DOI: 10.1207/S15328007SEM0904_8

[47] Nevitt, J., & Hancock, G. (2001). Performance of bootstrapping approaches to model test statistics and parameter standard error estimation in structural equation modeling. Structural Equation Modeling: A Multidisciplinary Journal, 8(3), 353–377. DOI: 10.1207/S15328007SEM0803_2

[48] Nigg, J. T. (2000). On inhibition/disinhibition in developmental psychopathology: Views from cognitive and personality psychology and a working inhibition taxonomy. Psychological Bulletin, 126(2), 220–246. DOI: 10.1037/0033-2909.126.2.220 10748641

[49] Nigg, J. T. (2001). Is ADHD a disinhibitory disorder? Psychological Bulletin, 127(5), 571–598. DOI: 10.1037/0033-2909.127.5.571 11548968

[50] Nigg, J. T., Wong, M. M., Martel, M. M., Jester, J. M., Puttler, L. I., Glass, J. M., Adams, K. M., Fitzgerald, H. E., & Zucker, R. A. (2006). Poor response inhibition as a predictor of problem drinking and illicit drug use in adolescents at risk for alcoholism and other substance use disorders. Journal of the American Academy of Child & Adolescent Psychiatry, 45(4), 468–475. DOI: 10.1097/01.chi.0000199028.76452.a9 16601652

[51] Noreen, S., & MacLeod, M. D. (2015). What do we really know about cognitive inhibition? Task demands and inhibitory effects across a range of memory and behavioural tasks. PLOS ONE, 10(8), e0134951 DOI: 10.1371/journal.pone.0134951 26270470 PMC4536050

[52] Pasolunghi, M. C., Cornoldi, C., & De Liberto, S. (1999). Working memory and intrusions of irrelevant information in a group of specific poor problem solvers. Memory & Cognition, 27(5), 779–790. DOI: 10.3758/BF03198531 10540807

[53] Rabbitt, P. (1997). Introduction: Methodologies and models in the study of executive function In Methodology of frontal and executive function (pp. 1–37). Routledge.

[54] Rey-Mermet, A., Gade, M., & Oberauer, K. (2018). Should we stop thinking about inhibition? Searching for individual and age differences in inhibition ability. Journal of Experimental Psychology: Learning, Memory, and Cognition, 44(4), 501–526. DOI: 10.1037/xlm0000450 28956944

[55] Rouder, J. N., & Haaf, J. M. (2019). A psychometrics of individual differences in experimental tasks. Psychonomic Bulletin & Review, 26(2), 452–467. DOI: 10.3758/s13423-018-1558-y 30911907

[56] Schmidt, C., Collette, F., Cajochen, C., & Peigneux, P. (2007). A time to think: Circadian rhythms in human cognition. Cognitive Neuropsychology, 24(7), 755–789. DOI: 10.1080/02643290701754158 18066734

[57] Schönbrodt, F. D., & Perugini, M. (2013). At what sample size do correlations stabilize? Journal of Research in Personality, 47(5), 609–612. DOI: 10.1016/j.jrp.2013.05.009

[58] Shilling, V. M., Chetwynd, A., & Rabbitt, P. M. A. (2002). Individual inconsistency across measures of inhibition: An investigation of the construct validity of inhibition in older adults. Neuropsychologia, 40(6), 605–619. DOI: 10.1016/S0028-3932(01)00157-9 11792402

[59] Simmons, J. P., Nelson, L. D., & Simonsohn, U. (2012). A 21 Word Solution. SSRN Electronic Journal. DOI: 10.2139/ssrn.2160588

[60] Singh, K., Ecker, U., Gignac, G., Brydges, C., & Rey-Mermet, A. (2018). Interference control in working memory. PsyArXiv. DOI: 10.31234/osf.io/fjrnq PMC7710115 33264336

[61] Soper, D. S. (2018). A-priori sample size calculator for structural equation models. http://www.danielsoper.com/statcalc

[62] Stahl, C., Voss, A., Schmitz, F., Nuszbaum, M., Tüscher, O., Lieb, K., & Klauer, K. C. (2014). Behavioral components of impulsivity. Journal of Experimental Psychology: General, 143(2), 850–886. DOI: 10.1037/a0033981 23957282

[63] Tabibnia, G., Monterosso, J. R., Baicy, K., Aron, A. R., Poldrack, R. A., Chakrapani, S., Lee, B., & London, E. D. (2011). Different forms of self-control share a neurocognitive substrate. Journal of Neuroscience, 31(13), 4805–4810. DOI: 10.1523/JNEUROSCI.2859-10.2011 21451018 PMC3096483

[64] Tiego, J., Testa, R., Bellgrove, M. A., Pantelis, C., & Whittle, S. (2018). A Hierarchical Model of Inhibitory Control. Frontiers in Psychology, 9 DOI: 10.3389/fpsyg.2018.01339 PMC6085548 30123154

[65] Townsend, J., & Ashby, F. G. (1983). The stochastic modeling of elementary psychological processes. Cambridge University Press.

[66] van der Sluis, S., de Jong, P. F., & van der Leij, A. (2007). Executive functioning in children, and its relations with reasoning, reading, and arithmetic. Intelligence, 35(5), 427–449. DOI: 10.1016/j.intell.2006.09.001

[67] van Velzen, L. S., Vriend, C., de Wit, S. J., & van den Heuvel, O. A. (2014). Response inhibition and interference control in obsessive-compulsive spectrum disorders. Frontiers in Human Neuroscience, 8 DOI: 10.3389/fnhum.2014.00419 PMC4052433 24966828

[68] Vandierendonck, A. (2017). A comparison of methods to combine speed and accuracy measures of performance: A rejoinder on the binning procedure. Behavior Research Methods, 49(2), 653–673. DOI: 10.3758/s13428-016-0721-5 26944576

[69] Westerhausen, R., Kompus, K., & Hugdahl, K. (2011). Impaired cognitive inhibition in schizophrenia: A meta-analysis of the Stroop interference effect. Schizophrenia Research, 133(1–3), 172–181. DOI: 10.1016/j.schres.2011.08.025 21937199

[70] Westland, C. J. (2010). Lower bounds on sample size in structural equation modeling. Electronic Commerce Research and Applications, 9(6), 476–487. DOI: 10.1016/j.elerap.2010.07.003

[71] Wickelgren, W. A. (1977). Speed-accuracy tradeoff and information processing dynamics. Acta Psychologica, 41(1), 67–85. DOI: 10.1016/0001-6918(77)90012-9

[72] Wilcox, R. R., & Keselman, H. J. (2003). Modern robust data analysis methods: Measures of central tendency. Psychological Methods, 8(3), 254–274. DOI: 10.1037/1082-989X.8.3.254 14596490

[73] Wolff, M., Krönke, K.-M., Venz, J., Kräplin, A., Bühringer, G., Smolka, M. N., & Goschke, T. (2016). Action versus state orientation moderates the impact of executive functioning on real-life self-control. Journal of Experimental Psychology: General, 145(12), 1635–1653. DOI: 10.1037/xge0000229 27736135

[74] Young, S. E., Friedman, N. P., Miyake, A., Willcutt, E. G., Corley, R. P., Haberstick, B. C., & Hewitt, J. K. (2009). Behavioral disinhibition: Liability for externalizing spectrum disorders and its genetic and environmental relation to response inhibition across adolescence. Journal of Abnormal Psychology, 118(1), 117–130. DOI: 10.1037/a0014657 19222319 PMC2775710

